# DEPTOR-mTOR Signaling Is Critical for Lipid Metabolism and Inflammation Homeostasis of Lymphocytes in Human PBMC Culture

**DOI:** 10.1155/2017/5252840

**Published:** 2017-02-27

**Authors:** Qi-bing Xie, Yan Liang, Min Yang, Yuan Yang, Xiao-min Cen, Geng Yin

**Affiliations:** ^1^Department of Rheumatology and Immunology, West China Hospital of Sichuan University, Chengdu 610041, China; ^2^Department of General Practice, West China Hospital of Sichuan University, Chengdu 610041, China

## Abstract

Abnormal immune response of the body against substances and tissues causes autoimmune diseases, such as polymyositis, dermatomyositis, and rheumatoid arthritis. Irregular lipid metabolism and inflammation may be a significant cause of autoimmune diseases. Although much progress has been made, mechanisms of initiation and proceeding of metabolic and inflammatory regulation in autoimmune disease have not been well-defined. And novel markers for the detection and therapy of autoimmune disease are urgent. mTOR signaling is a central regulator of extracellular metabolic and inflammatory processes, while DEP domain-containing mTOR-interacting protein (DEPTOR) is a natural inhibitor of mTOR. Here, we report that overexpression of DEPTOR reduces mTORC1 activity in lymphocytes of human peripheral blood mononuclear cells (PBMCs). Combination of DEPTOR overexpression and mTORC2/AKT inhibitors effectively inhibits lipogenesis and inflammation in lymphocytes of PBMC culture. Moreover, DEPTOR knockdown activates mTORC1 and increases lipogenesis and inflammations. Our findings provide a deep insight into the relationship between lipid metabolism and inflammations via DEPTOR-mTOR pathway and imply that DEPTOR-mTOR in lymphocytes of PBMC culture has the potential to be as biomarkers for the detection and therapies of autoimmune diseases.

## 1. Introduction

Autoimmune diseases, which are defined by abnormal immune response of the body against substances and tissues normally present in the body, increase the risk of developing multiple disorders [1–3]. Polymyositis [4, 5], dermatomyositis [6, 7], and rheumatoid arthritis [3, 8] are typical autoimmune diseases in modern society. For example, polymyositis is a chronic illness featuring progressive muscle weakness with periods of increased symptoms, including inflammation of the muscles or associated tissues [9, 10]. So far, the major understanding of pathophysiology in autoimmune diseases has been the irregular immunity and inflammation of immune cells [11, 12]. Based on this point, suppressive drugs are necessary to decrease the immune response and inflammation in the treatment of autoimmune diseases. Therefore, it is important to elucidate mechanisms of initiation and proceeding of inflammatory regulation in immune cells for autoimmune disease treatment.

Notably, mTOR signaling senses extracellular stimulations and regulates many biological processes including inflammations [13, 14]. The mechanistic target of rapamycin (mTOR) is a phosphatidylinositol 3-kinase- (PI3K-) like serine/threonine protein kinase that is evolutionarily conserved in all eukaryotes [15, 16]. Deregulation of mTOR signaling has been shown that it is closely associated with cancers and metabolic diseases as well as autoimmune diseases. mTOR resides in two distinct complexes referred to as mTOR complex 1 (mTORC1) and mTOR complex 2 (mTORC2) [17]. mTORC1 comprises mTOR, Raptor, DEPTOR, mLST8, and PRAS40, while mTORC2 comprises mTOR with Rictor, mSIN1, DEPTOR, mLST8, and Protor [13]. Interestingly, it is identified that DEP domain-containing mTOR-interacting protein (DEPTOR) directly interacts with both mTORC1 and mTORC2 complexes [18]. Ectopic high DEPTOR expression decreases mTORC1 activity and S6K1-mediated feedback loop on PI3K/AKT to regulate cell metabolism and survival [18]. Thus, DEPTOR is even accepted as a natural endogenous mTORC1 inhibitor.

All through the regulation of inflammations, mTOR signaling modulates levels of inflammatory cytokines produced by immune cells, whereas mTOR (especially for mTORC1) is a master regulator of cell metabolism, such as protein synthesis, lipid biosynthesis (lipogenesis), and glucose oxidation [19]. Importantly, the SREBPs are key factors transcriptionally regulated by mTORC1, which stimulates the expression of genes encoding nearly all of these lipogenic enzymes [20]. Nowadays, it is appreciated that mTORC1 controls lipid homeostasis both physiologically and pathologically. On the other hand, synthesized free fatty acids (FFAs) are well-characterized factor for causing production of inflammatory factors [21, 22]. Hence, it is proposed that mTORC1 signaling may control inflammatory reactions via metabolic alternations.

Previous studies have reported that TNF-*α*, IL-6, and NF-*κ*B are increased in nutrition-enriched conditions, and this increase is correlated with lipid-metabolic disorders [23–25]. Although these studies demonstrate lipid metabolism may correlate with inflammation, clear mechanisms of how lipid metabolism is coupled with inflammation in immune cells are not well defined. Here, we report that mTOR signaling is a central regulator of lipid metabolism-mediated inflammation in lymphocytes of human PBMC culture. Overexpression of mTORC1 inhibitor DEPTOR partly reduces mTORC1 activity but does not decrease lipogenesis and inflammation. We assume this may be due to activated mTORC2 pathway. Addition of AKT inhibitors decreases lipogenesis and inflammation in DEPTOR overexpressed cells. Moreover, DEPTOR knockdown activates mTORC1 and increases lipogenesis and inflammation. Our findings will help to provide a deep insight into lipid metabolism and inflammations coupling via DEPTOR-mTOR pathway and imply that DEPTOR-mTOR in lymphocytes of PBMC culture has the potential to be biomarkers for the detection and therapies of autoimmune diseases.

## 2. Materials and Methods

### 2.1. Chemicals and Reagents

RPMI-1640 and Fetal Bovine Serum (FBS) for PBMC culture were purchased from GIBCO Invitrogen (Carlsbad, CA, USA). The DEPTOR, mTOR, pp70S6K (Thr389), p70S6K, p4EBP1 (Thr37/46), 4EBP1, pAKT (Ser473), AKT, and beta-actin antibodies were all from Cell Signaling Technology (Danvers, MA, USA). To knockdown endogenous DEPTOR, shRNA targeting sequence of DEPTOR gene and a scramble shRNA (NC) were synthesized. shRNA-encoding plasmids were cotransfected with the Delta VPR envelope and CMV VSV-G packaging plasmids into growing lymphocytes of PBMC culture. Lentiviral shRNAs to human DEPTOR were previously described, which are named with the numbers found at the TRC public website: human DEPTOR_1 shRNA: TRCN0000110157; NM_145470.1-1164s1c1; human DEPTOR_2 shRNA: TRCN0000110159; NM_145470.1-1165s1c1 [18]. For DEPTOR overexpression, the myc-tagged DEPTOR construct was generated by subcloning the PCR-amplified human DEPTOR coding sequence into pRK5-myc vectors for further lentiviral transfections. Other chemicals were of the highest purity available.

### 2.2. Ethical Approval and Cell Cultures

Healthy volunteers for PBMC cutlure were provided written informed consent approved by the Ethics Administration Office of West China Hospital, Sichuan University. For the preparation of PBMC culture, cells were extracted from heparinized whole blood by differential centrifugation by Histopaque 1077 (Sigma) as instructions. For Western blots and real-time PCR experiments, cells were plated in 6-well plates at 1.0 × 10^6^ cells/mL. The cells were incubated in RPMI-1640 medium containing 10% FBS plus antibiotics for 24 hours in 5% CO_2_ at 37°C. After culturing, the cells were harvested for subsequent examinations.

### 2.3. Lysates Preparation and Western Blots

For Western blots, prepared cells were trypsinized and harvested, washed with PBS once, and resuspended in cell lysis buffer (PBS with 1% Triton X-100 and protease inhibitors). After brief sonication, cell lysates were centrifuged at 13,000 rpm for 5 min. Protein concentration was determined so that equivalent amounts of lysate based on protein concentration were added to an equal volume of 2X Laemmli buffer and boiled for 10 min. For Western blot analysis, the procedure was according to standard protocols. Finally, proteins were detected by Super Signal® enhanced chemiluminescence development (ECL) (Thermo Scientific Pierce) reagent and exposed to films (Kodak). The protein level quantification was carried out by ImageJ.

### 2.4. Quantitative Real-Time PCR

Total RNA was extracted from tissues using Trizol reagent (Invitrogen). RNA was subjected to reverse transcription with reverse transcriptase as per manufacturer's instructions (Fermentas). Quantitative real-time PCR was performed using the Bio-Rad iQ5 system, and the relative gene expression was normalized to internal control as beta-actin. Primer sequences for SYBR Green probes of target genes are as shown in Table 1.

### 2.5. Statistical Analysis

Data represent the mean and standard error of the mean (SEM). ANOVA tests for comparisons were performed for all statistical significance analysis using GraphPad Prism software. ^*∗*^*P* < 0.05, ^*∗∗*^*P* < 0.01, and ^*∗∗∗*^*P* < 0.001.

## 3. Results

### 3.1. Overexpressed DEPTOR Decreases mTORC1 and Increases mTORC2 Activity

**Figure 1:**
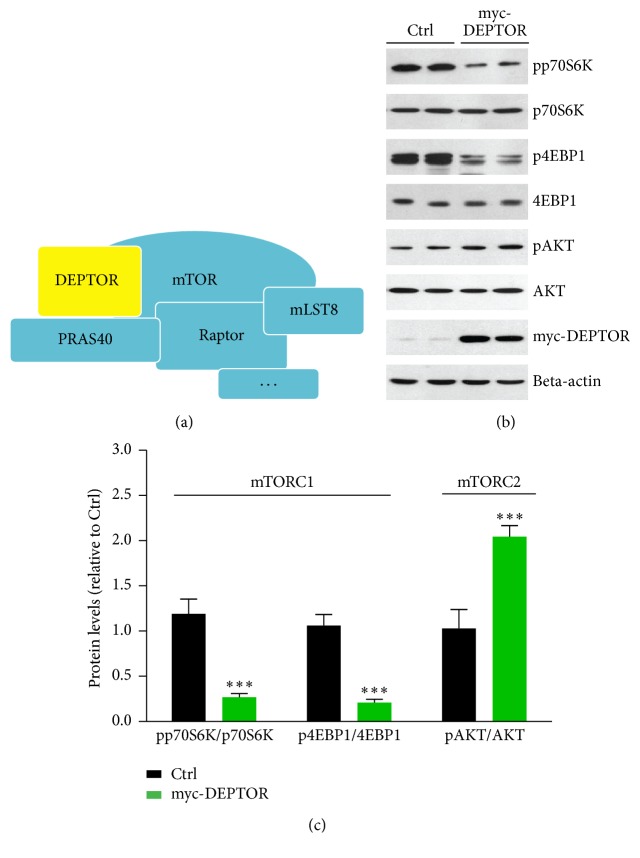
Overexpressed DEPTOR decreases mTORC1 and increases mTORC2 activity. (a) A schematic model showed the components of mTORC1 complexes. Raptor is the characterized adaptor protein of mTORC1, and mTOR works as the kinase to phosphorylate downstream substrates. DEPTOR is the natural inhibitor of mTORC1. (b-c) Western blots and quantifications showed that mTORC1 activity, indicated by phosphorylations of p70S6K and 4EBP1, was downregulated by myc-DEPTOR overexpression in lymphocytes of PBMC culture. Results are averages of three independent experiments. Data represent mean ± SEM. ^*∗∗∗*^*P* < 0.001.

DEPTOR is a natural inhibitor of mTOR via directly binding to both mTORC1 and mTORC2 (Figure 1(a)). Previous studies identify that DEPTOR depletion activates mTORC1 and mTORC2 signaling in several cell and animal models [18, 26]. Moreover, overexpression of DEPTOR inhibits mTORC1 and further activates PI3K/AKT signaling [18]. However, how DEPTOR regulates lymphocyte mTOR activity is not well defined. Thus, overexpression of DEPTOR and mTORC1/2 activity in lymphocytes of PBMC culture were firstly analyzed. Biochemical results showed that protein levels of markers of mTORC1 pathway (pp70S6K and p4EBP1) [27] were both decreased by DEPTOR overexpression (Figures 1(b) and 1(c)). On the other hand, it is noted that mTORC2 activity, indicated by phospho-AKT, is increased by DEPTOR overexpression in lymphocytes of PBMC culture (Figures 1(b) and 1(c)). Therefore, our results suggest that overexpressed DEPTOR decreases mTORC1 but increases mTORC2 activity, which may affect downstream lipid metabolism inflammations.

### 3.2. Overexpressed DEPTOR Does Not Inhibit Lipogenesis and Inflammation in Lymphocytes of PBMC Culture

**Figure 2:**
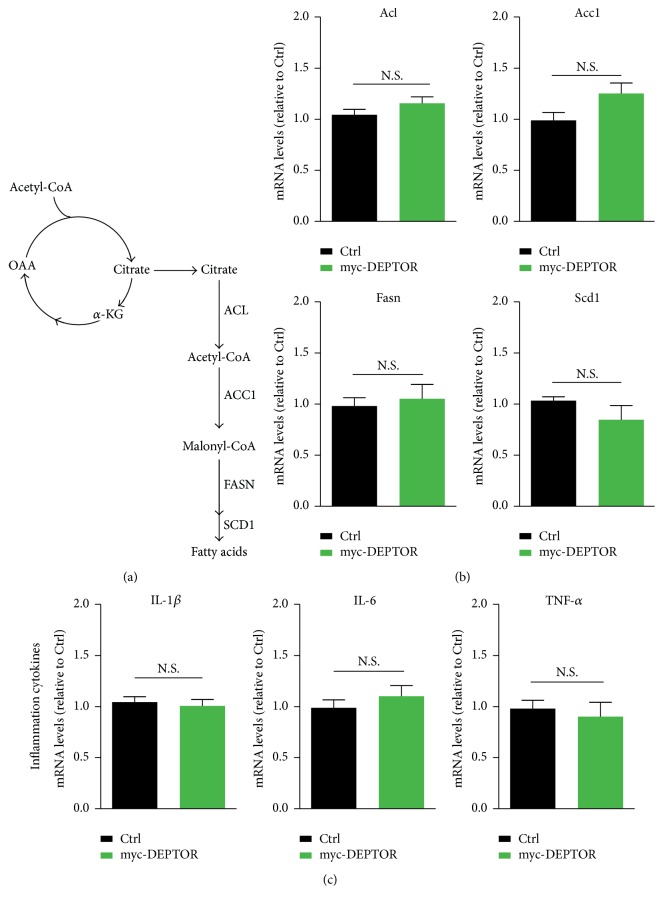
Overexpressed DEPTOR does not inhibit lipogenesis and inflammation. (a) A schematic model showed the signaling of mTORC1-controlled lipogenesis. ACL, ACC1, FASN, and SCD1 were downstream enzymes in fatty acid synthesis. (b) Real-time RCR results showed that mRNA levels of lipogenesis genes, including Acl, Acc1, Fasn, and Scd1, were not reduced in DEPTOR overexpressed cells. Results are averages of three independent experiments. Data represent mean ± SEM. N.S.: no statistical significance. (c) Real-time RCR results showed that mRNA levels of inflammation genes, including IL1-*β*, IL-6, and TNF-*α*, were not reduced in DEPTOR overexpressed cells. Results are averages of three independent experiments. Data represent mean ± SEM. N.S.: no statistical significance.

Considering that mTOR pathway is a central regulator of lipid metabolism and inflammatory homeostasis [13], we further investigated whether DEPTOR overexpression would like to alter lipogenesis and inflammation in lymphocytes of PBMC culture. The expression of lipogenesis enzymes is controlled by the SREBP1 transcription factor, which is tightly controlled by mTORC1 [20] (Figure 2(a)). By real-time PCR assays, we found that the expression of lipid metabolism genes, in particular Acl, Acc1, Fasn, and Scd1, was not dramatically reduced by DEPTOR overexpression (Figure 2(b)). Since lipids are well-characterized factor for causing production of inflammatory factors, we further examined the expression of inflammation factors, including IL1-*β*, IL-6, and TNF-*α* [28]. The results of quantitative real-time PCR showed that the expressions of all these three inflammation factors are consistently not dramatically altered by DEPTOR overexpression (Figure 2(c)). Taken together, all these results indicate that inhibition of mTORC1 activity via DEPTOR may not inhibit lipogenesis and inflammation in lymphocytes of PBMC culture.

### 3.3. AKT Inhibitors Downregulate Lipogenesis and Inflammation in DEPTOR Overexpressed Cells

**Figure 3:**
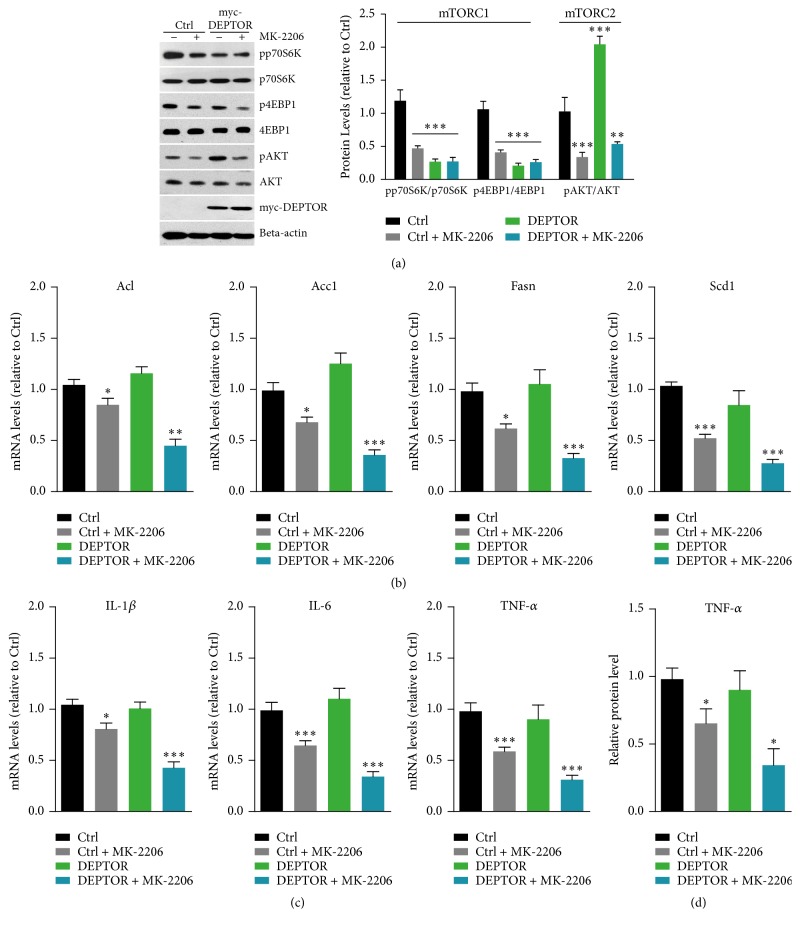
AKT inhibitors downregulate lipogenesis and inflammation in DEPTOR overexpressed cells. (a) Western blots and quantifications showed that mTORC1 activity, indicated by phosphorylations of p70S6K and 4EBP1, was downregulated by myc-DEPTOR overexpression and MK-2206 (1 *μ*M for 4 hours, same as follows) conditions. However, mTORC2 activity (indicated by pAKT/AKT) was blocked by MK-2206 treatment in lymphocytes of PBMC culture. Results are averages of three independent experiments. Data represent mean ± SEM. Data represent mean ± SEM. ^*∗∗*^*P* < 0.01 and ^*∗∗∗*^*P* < 0.001. (b) Real-time RCR results showed that mRNA levels of lipogenesis genes, including Acl, Acc1, Fasn, and Scd1, were dramatically reduced in MK-2206 treated DEPTOR overexpressed cells. Results are averages of three independent experiments. Data represent mean ± SEM. Data represent mean ± SEM. ^*∗*^*P* < 0.05, ^*∗∗*^*P* < 0.01, and ^*∗∗∗*^*P* < 0.001. (c) Real-time RCR results showed that mRNA levels of inflammation genes, including IL1-*β*, IL-6, and TNF-*α*, were dramatically reduced in MK-2206 treated DEPTOR overexpressed cells. Results are averages of three independent experiments. Data represent mean ± SEM. ^*∗*^*P* < 0.05 and ^*∗∗∗*^*P* < 0.001. (d) ELISA results showed that protein levels of TNF-*α* were decreased in MK-2206 treated DEPTOR overexpressed cells. Results are averages of three independent experiments. Data represent mean ± SEM. ^*∗*^*P* < 0.05.

Our results support the fact that mTORC1 inactivation by DEPTOR may not inhibit lipogenesis and inflammation in lymphocytes of PBMC culture. Thus, we propose that increased mTORC2 may be responsible for the maintained lipid and inflammation homeostasis [29]. We examined the lipogenesis and inflammation factors in AKT-inhibited DEPTOR overexpressed cells. MK-2206 is a well-defined AKT inhibitor and inhibits autophosphorylation of AKT at T308 and S473 sites [30]. We applied MK-2206 to DEPTOR overexpressed cells and examined downstream signaling. Biochemical results showed that, as MK-2206 treatment, the mTORC1 and AKT activities were decreased, indicated by pp70S6K, p4EBP1, and pAKT (Figure 3(a)).

Accordingly, we further examined the lipogenesis and inflammation in these AKT-inhibited and DEPTOR overexpressed cells. As expected, real-time PCR results showed that expressions of lipogenesis genes, Acl, Acc1, Fasn, and Scd1, were dramatically reduced by the overexpression of DEPTOR in lymphocytes of PBMC culture (Figure 3(b)). Consistently, we found that expressions of inflammation factors, including IL1-*β*, IL-6, and TNF-*α*, were also reduced by MK-2206 treatment (Figure 3(c)). Moreover, we found that the protein level of TNF-*α* was consistently decreased by MK-2206 (Figure 3(d)). All these facts suggest that AKT inhibitors decrease mTORC1/2 signaling and lipogenesis and inflammation in DEPTOR overexpressed lymphocytes of PBMC culture.

### 3.4. DEPTOR Knockdown Increases mTORC1 Activity, Lipogenesis, and Inflammation in Lymphocytes of PBMC Culture

**Figure 4:**
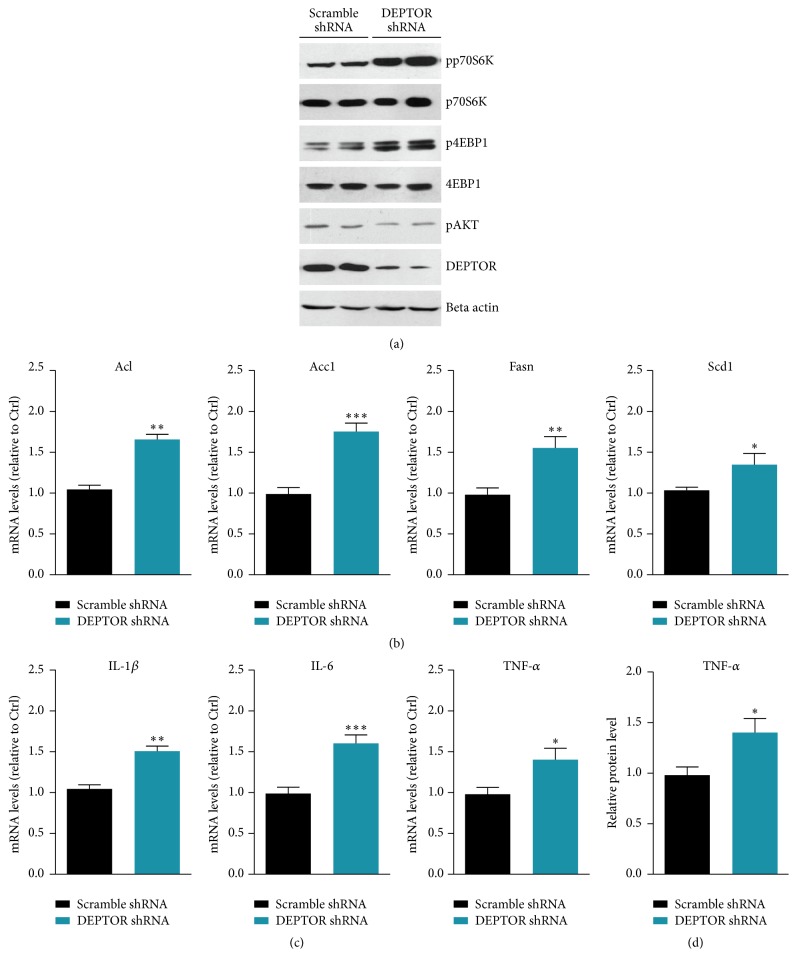
DEPTOR knockdown increases mTORC1 activity, lipogenesis, and inflammation in lymphocytes of PBMC culture. (a) Western blots showed that mTORC1 activity, indicated by phosphorylations of p70S6K and 4EBP1, was upregulated by DEPTOR shRNA in lymphocytes of PBMC culture. Noted that pAKT was slightly downregulated by DEPTOR knockdown. (b) Real-time RCR results showed that mRNA levels of lipogenesis genes, including Acl, Acc1, Fasn, and Scd1, were increased in DEPTOR knockdown cells. Results are averages of three independent experiments. Data represent mean ± SEM. ^*∗*^*P* < 0.05, ^*∗∗*^*P* < 0.01, and ^*∗∗∗*^*P* < 0.001. (c) Real-time RCR results showed that mRNA levels of inflammation genes, including IL1-*β*, IL-6, and TNF-*α*, were increased in DEPTOR knockdown cells. Results are averages of three independent experiments. Data represent mean ± SEM. ^*∗*^*P* < 0.05, ^*∗∗*^*P* < 0.01, and ^*∗∗∗*^*P* < 0.001. (d) ELISA results showed that protein level of TNF-*α* was increased in DEPTOR knockdown cells. Results are averages of three independent experiments. Data represent mean ± SEM. ^*∗*^*P* < 0.05.

Since overexpression of DEPTOR has been found to inactivate mTORC1 pathway, we proposed that knockdown DEPTOR activity may activate mTORC1 and downstream lipogenesis and inflammations. To test this hypothesis, we carried out DEPTOR RNAi to inhibit DEPTOR expression and investigated the mTORC1 signaling alternations. Biochemical results showed that DEPTOR knockdown could increase mTORC1 activity (indicated by p70S6K and 4EBP1 phosphorylations) and slightly decrease mTORC2/AKT activity (Figure 4(a)). Based on this point, we further examined the lipogenesis and inflammation in DEPTOR knockdown cells. Results showed that expressions of lipogenesis genes, Acl, Acc1, Fasn, and Scd1, were increased by DEPTOR knockdown in lymphocytes of PBMC culture (Figure 4(b)). Moreover, inflammation factors, including IL1-*β*, IL-6, and TNF-*α*, were also increased by DEPTOR knockdown (Figure 4(c)). Moreover, we found that the protein level of TNF-*α* was consistently increased by DEPTOR knockdown (Figure 4(d)). Collectively, these data indicate that DEPTOR knockdown increases mTORC1 activity, lipogenesis, and inflammation.

## 4. Discussion

**Figure 5:**
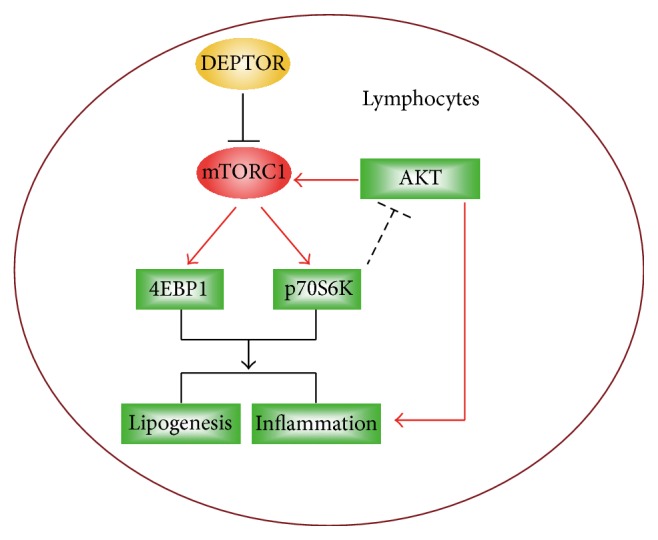
Schematic representation explaining the network of DEPTOR, mTORC1, mTORC2, and lipogenesis/inflammation networks. DEPTOR is a natural inhibitor of mTORC1 pathway. Overexpression of mTORC1 inhibitor DEPTOR may partly reduce mTORC1 activity but may not decrease lipogenesis and inflammation. Addition of AKT inhibitors may decrease lipogenesis and inflammation in DEPTOR overexpressed cells. Moreover, DEPTOR knockdown may activate mTORC1 and increase lipogenesis and inflammation.

The balance of metabolic and inflammatory events is critical for lymphocyte homeostasis. However, the irregular lipid metabolism and ectopic inflammation may be a direct cause of autoimmune diseases, such as polymyositis, dermatomyositis, and rheumatoid arthritis [1, 6, 12]. In the present study, we find that DEPTOR, a natural inhibitor of mTOR, may regulate lipid homeostasis and inflammation in lymphocytes of PBMC culture. We show that overexpression of DEPTOR may partly reduce mTORC1 activity but may not decrease lipogenesis and inflammation. This is due to activated mTORC2 pathway and addition of AKT inhibitors may decrease lipogenesis and inflammations. Oppositely, DEPTOR knockdown activates mTORC1 and increases lipogenesis and inflammation in lymphocytes of PBMC culture. Our findings indicate that DEPTOR-mTOR signaling is critical for homeostasis of metabolic and inflammations and suggest that DEPTOR-mTOR signaling to be markers of autoimmune diseases (Figure 5).

Nowadays, it has been widely accepted that DEPTOR interacts with mTORC1 [18]. And DEPTOR regulates downstream metabolic events via mTORC1/2 signaling. For example, DEPTOR acts as a tumor suppressor by blocking mTORC1 activity to inhibit protein synthesis and cell proliferation [31]. DEPTOR expression can reduce mTORC1 activity and S6K1-mediated feedback inhibition of PI3K/AKT pathways, which contributes to the cell survival [32]. Therefore, the expression of intracellular DEPTOR must be precisely regulated. In our studies, we noted that the overexpression of DEPTOR indeed inhibits mTORC1 activity but failed to block lipogenesis and inflammation as our expectations. We demonstrate that increased mTORC2/AKT pathway may be responsible for this “resistance.” Addition of AKT inhibitor MK-2206 may enhance the effect of DEPTOR on inactivation of lipogenesis and inflammation in lymphocytes of PBMC culture. Therefore, DEPTOR seems to act as a key factor for the balance of mTORC1 and mTORC2 pathways, especially in metabolic and inflammatory regulations.

mTOR pathway is a master regulator of cell metabolism and inflammations [13]. In 2008, a hallmark paper demonstrates that mTORC1 inhibition blocks expressions of genes involved in lipogenesis and impairs the nuclear accumulation of the SREBPs [33]. Subsequent studies confirm this finding and figure out that mTORC2/AKT may regulate lipogenesis in mTORC1 dependent and independent manners [29]. Recently, it is reported that mTORC1 phosphorylates CRTC2 and attenuates its inhibitory effect on COPII-dependent SREBP1 maturation and downstream lipogenesis [34]. All these studies clearly show how mTORC1/2 pathway modulates lipid metabolism. However, the biological functions of mTOR-lipid metabolism in inflammatory reactions and immune systems have been not well defined. In previous studies, we demonstrate that mTORC1 pathway is critical for the initiation of inflammatory reactions in synovial cells [35]. Moreover, lipid peroxidation-mediated inflammation promotes cell apoptosis through activation of NF-*κ*B pathway in rheumatoid arthritis synovial cells [28]. In the present study, we intended to study upstream regulators of mTOR pathway and its metabolic function in inflammatory reactions. Our data define DEPTOR, a well-known mTOR inhibitor, that is a critical mediator of lipogenesis and inflammation in lymphocytes of PBMC culture. The next question would be to clarify pathological functions of DEPTOR in autoimmune diseases and to design targeted drugs.

## 5. Conclusion

In conclusion, the present findings supported the fact that DEPTOR-mTOR signaling is a central regulator of lipid metabolism-mediated inflammation in lymphocytes of PBMC culture. Combination of DEPTOR overexpression and mTORC2/AKT inhibitors may effectively inhibit lipogenesis and inflammation. Moreover, DEPTOR knockdown may activate mTORC1 and increase lipogenesis and inflammations. Our findings provide a deep insight into lipid metabolism and inflammations coupling via DEPTOR-mTOR pathway and imply that DEPTOR-mTOR of lymphocytes of PBMC culture has potential to be markers for the detection and therapies of autoimmune diseases.

## Figures and Tables

**Table 1:** Primer sequences for SYBR Green probes of target genes.

Acl	F: 5′-GAAGCTGACCTTGCTGAACC-3′
	R: 5′-CTGCCTCCAATGATGAGGAT-3′

Acc1	F: 5′-TCTTTTCCTCGGAGCATGACA-3′
	R: 5′-GACCTCTCTACTCACTTCTCCAG-3′

Fasn	F: 5′-GGAGGTGGTGATAGCCGGTAT-3′
	R: 5′-TGGGTAATCCATAGAGCCCAG-3′

Scd1	F: 5′-TTTCGAAGACGTCAGAGTGC-3′
	R: 5′-TGCGACTGTAGGTCTGGTTC-3′

Il-1*β*	F: 5′-CTGGTGTGTGACGTTCCCATTA-3′
	R: 5′-CCGACAGCACGAGGCTTT-3′

Il-6	F: 5′-TTCCATCCAGTTGCCTTCTTG-3′
	R: 5′-TGGGAGTGGTATCCTCTGTGA-3′

Tnf-*α*	F: 5′-CATCTTCTCAAAATTCGAGTGACA-3′
	R: 5′-TGGGAGTAGACAAGGTACAACCC-3′

*β*-Actin	F: 5′-TCCATCATGAAGTGTGACGT-3′
	R: 5′-TACTCCTGCTTGCTGATCCAC-3′
